# The Concept, Practice, Application, and Results of Locally Based Monitoring of the Environment

**DOI:** 10.1093/biosci/biab021

**Published:** 2021-04-28

**Authors:** Finn Danielsen, Martin Enghoff, Michael K Poulsen, Mikkel Funder, Per M Jensen, Neil D Burgess

**Affiliations:** Nordic Foundation for Development and Ecology, Copenhagen, Denmark; Nordic Foundation for Development and Ecology, Copenhagen, Denmark; Nordic Foundation for Development and Ecology, Copenhagen, Denmark; Danish Institute of International Studies, also in Copenhagen; Faculty of Science, University of Copenhagen; Centre for Macroecology, Evolution, and Climate, University of Copenhagen and with United Nations Environment Programme's World Conservation Monitoring Centre, Cambridge, United Kingdom

## Abstract

Locally based monitoring is typically undertaken in areas in which communities have a close attachment to their natural resource base. We present a summary of work to develop a theoretical and practical understanding of locally based monitoring and we outline tests of this approach in research and practice over the past 20 years. Our tests show that locally based monitoring delivers credible data at local scale independent of external experts and can be used to inform local and national decision making within a short timeframe. We believe that monitoring conducted by and anchored in communities will gain in importance where scientist-led monitoring is sparse or too expensive to sustain and for ecosystem attributes in cases in which remote sensing cannot provide credible data. The spread of smartphone technology and online portals will further enhance the importance and usefulness of this discipline.

It is increasingly recognized that local community members who may not qualify as experts in academic terms can be very knowledgeable about local resources (Zhao et al. 2016, Mustonen and Tossavainen 2018). Moreover, local community members have an important role in the sustainable use of natural resources (Garnett et al. 2018, O'Bryan et al. 2020). The development of technology and, in particular, mobile devices and social media, which have penetrated across the world (now also in developing regions), allows for hundreds of millions of people to participate in scientific processes and to gather information and obtain results that are both locally and globally relevant and potentially transformational in scope (https://ebird.org/home, www.inaturalist.org). Expanding the scientific base to include community members or citizen scientists would provide multiple, fine-scaled data points on the changes in the natural resources of the planet—allowing credible models to be built on variables that cannot be measured by remote sensing (Stephenson et al. 2015)—and in regions in which there are no scientifically trained personnel. Moreover, if the community members’ role is broadened from gathering data to also asking questions, identifying priorities, interpreting information, putting it into context, and communicating their findings and proposed management actions, the results can transform the understanding of how large scale environmental changes play out at the local level. Relevant measures that could be tracked include species abundance changes, habitat use and degradation, local use of biomass and hunted species, local pressures on nature, climate-moderated range shifts, and the introductions of species outside their normal ranges.

Over the past 20 years, we have worked together with communities, government agencies, and civil society organizations to enhance local capacity for the monitoring and management of natural resources. Research by ourselves and colleagues on local community member monitoring has appeared in a number of textbooks (Spellerberg 2005, Milner-Gulland and Rowcliffe 2007, Buckley 2009, Gardner 2010, McComb et al. 2010, Sodhi and Ehrlich 2010, Maschinski and Haskins 2012, Jones et al. 2013, Porter-Bolland et al. 2013, Lepczyk et al. 2020), and our findings have contributed to policy advancement, notably in the field of climate change (United Nations 2008, 2009). The work has facilitated the uptake of local knowledge into the 2019 Intergovernmental Science-Policy Platform on Biodiversity and Ecosystem Services global assessment (IPBES 2019). There are today locally based environmental monitoring programs on all the inhabited continents.

This review presents the main results of our research on locally based environmental monitoring. The analyses we present have been peer reviewed and published in the past. In the present article, we combine our tests of monitoring approaches and put them into a broader perspective. We outline how we developed a spectrum of natural resource monitoring systems, with varying degrees of (relative) contributions of local stakeholders and professional researchers. We elaborate on the types of data that locally based monitoring can generate, and we discuss the link from monitoring to decision-making and empowerment in natural resource management. Finally, we explore the opportunity to develop this kind of monitoring into the future.

## How did we get here?

**Figure 1.**
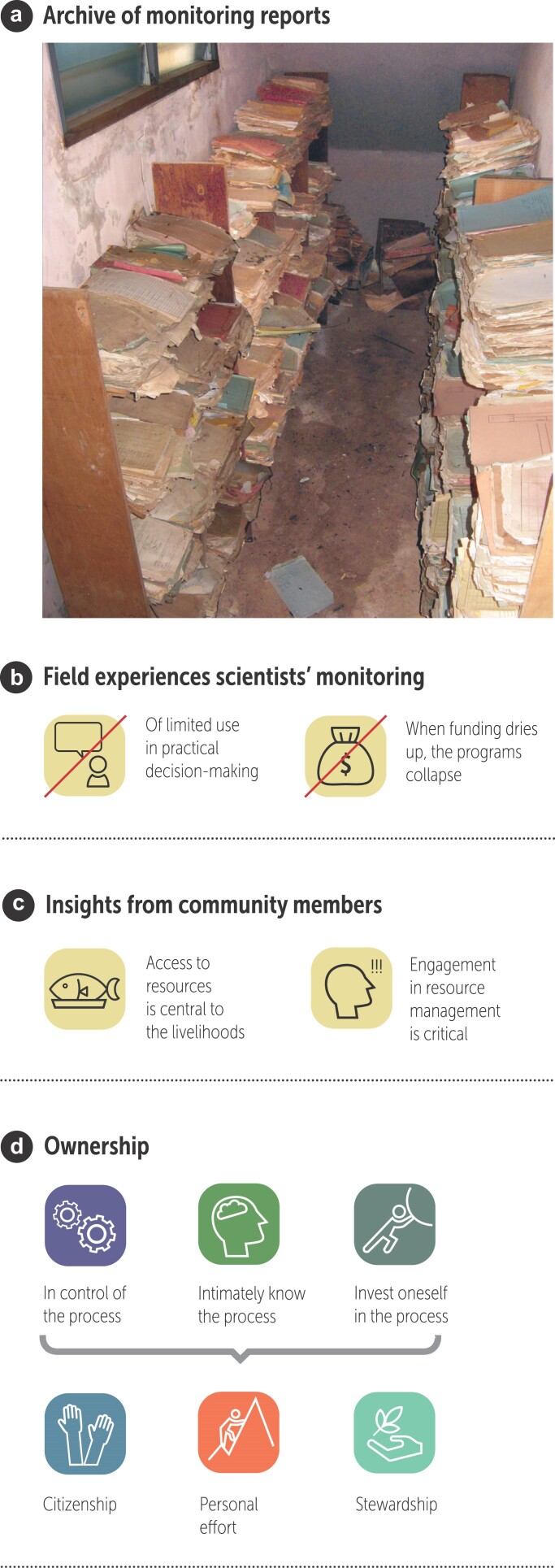
Issues for environmental monitoring. (a) Data storage: Deteriorating archive of biodiversity monitoring reports in a national park's headquarters in Ghana. (b), (c) Experiences from the field and from community members. (d) Potential links between community member involvement in and feelings of ownership of the management process. Photograph: A. Cole Burton.

Our interest in local community engagement in monitoring began when we realized the gap between ideals and realities with respect to monitoring systems. In many areas, monitoring reports piled up and were rarely used, except by termites (figure 1a; Burton 2012). Monitoring programs often were unable to contribute to natural resource management because they were ineffective in integrating information into decision-making (figure 1b; Danielsen et al. 2000, Sheil 2001). Moreover, many programs ceased to function when their financial support ended. For natural resource management purposes, monitoring needs to be targeted at information that delivers guidance. In-depth studies of how biological community structure and species richness are affected by different environmental changes rarely provide such information (Fox et al. 2017, Schuette et al. 2018). With these facts in mind, we led a call for an alternative approach to environmental monitoring in which the focus is on simple and cost-effective tools that seek to encourage the participation of local communities in natural resource management and that strengthen existing local systems for monitoring and managing natural resources (Danielsen et al. 2003).

Importantly, our interest in community engagement in monitoring also was informed by insights from community members (figure 1c). A community member was defined as a citizen living close to and using natural resources. For community members, it is relevant to spend time and efforts on natural resource monitoring if it addresses critical collective or individual needs. Access to natural resources is often a core component in the lives, livelihoods, and survival of communities, and engagement in the resource management process is therefore key to them (Funder et al. 2013, Brofeldt et al. 2018). Moreover, involvement in monitoring can enhance communal cohesion and provide a joint sense of purpose (Cundill and Fabricius 2010, Brown et al. 2012).

Community involvement in monitoring also is relevant because it may lead to ownership of the natural resource management process (Sterling et al. 2017, Marrocoli et al. 2018). People may develop sentiments of ownership for a process in three ways (Pierce and Jussila 2011): through having control over the process, having profound knowledge of the process, and investing oneself in the process (figure 1d). The effects stemming from ownership sentiments may include acts of citizenship, personal effort, and stewardship. There are however important exemptions—for instance, if the community members’ economic and social conditions and their relationship with the agencies having management responsibility are poor. Perceived ownership also may provide incentives for community members to protect their rights to the natural resources and to control elements that they may not be satisfied with (within the limitations provided by the overall policies and power relationships that frame the local situation; Matilainen et al. 2019). These considerations made us interested in locally based monitoring. Community members’ engagement in a natural resource monitoring and management process may have a triple win effect. It may simultaneously provide data of value to multiple users beyond the local, lead to social organization for monitoring and management, thereby enhancing the capacity of the community members, and contribute to knowledge generation at the local level about natural resources and resource management in general, and about local practices of resource use in particular. We now turn to how locally based monitoring differs from other forms of environmental monitoring.

## What is locally based monitoring?

**Figure 2.**
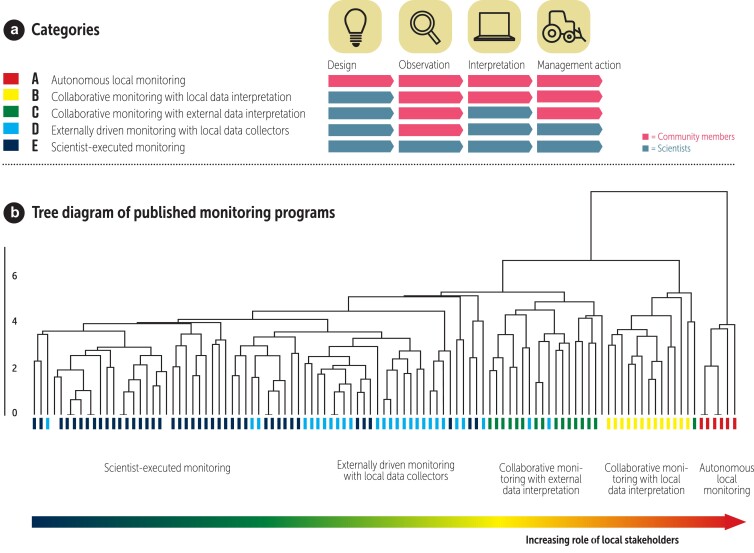
Spectrum of monitoring approaches with varying levels of involvement of citizens and scientists. (a) The five categories of monitoring systems, defined by the degree of participation in design, observation, interpretation, and management action. (b) Tree diagram of published environmental monitoring systems, based on a cluster analysis of 17 parameters among 107 monitoring systems, extracted from our review of approximately 3500 papers presenting published monitoring results. The relative role of local stakeholders in the monitoring systems increases from left to right between the five categories of monitoring systems. From Danielsen and colleagues (2009, 2014c), including the online data sets for panel (b).

Natural resource monitoring can be undertaken by both scientists and community members. Discussion of the relative benefits and disadvantages of locally based natural resource monitoring and that executed by professional researchers tends to be bimodal and is focused on these two extremes, but, in reality, these simply form the ends of a spectrum of possible monitoring protocols (figure 2a; Danielsen et al. 2009).

To examine this gradient more closely, we developed a typology of all monitoring systems, not just those that are locally based (Danielsen et al. 2014c). We reviewed the scientific literature for papers presenting published monitoring results and used a cluster analysis to segregate groups of monitoring systems with similar traits. The results are shown in the tree diagram in figure 2b. The lowest row of branches (the leaves) in the tree represents individual published monitoring systems. The height of each branch in the tree is proportional to the difference between the leaves. The color of the leaves represents the category of monitoring system that the leaves belong to. Our findings suggest that there are five categories, ranging from efforts in which monitoring is undertaken solely by professional researchers to entirely local efforts, with all the work undertaken by local people. The five categories are defined by the degree of scientist and local participation in system design, data collection, data interpretation, and implementation of management intervention (figure 2a). The most distinct (i.e., the most easily separable, those with the highest branches in figure 2b) monitoring systems were *Autonomous local monitoring* systems (category A). They have two unique characteristics: Local stakeholders took the initiative to set them up, and they are fully locally managed and resourced; scientists are not involved. These monitoring systems often use Local Ecological Knowledge (Archer et al. 2020, Camino et al. 2020) indicators and qualitative approaches. Examples are customary conservation regimes (Sheil et al. 2015, Tomaisini and Theilade 2019), and hunter, fishing, and farmer clubs’ monitoring of, for example, moose (*Alces alces*), bears (Ursus spp.), trout and salmon (Salmo spp.), and water flow in streams.

The second most distinct group is *Collaborative monitoring with local data interpretation* (category B). In these monitoring systems, scientists took the original initiative, but local stakeholders play a central role in asking questions, identifying priorities, and designing the systems, and they also collect, process, and interpret the data (Danielsen et al. 2014d). Examples are communities’ and rangers’ monitoring of resource use and wildlife (Brofeldt et al. 2018, Constantino 2020) and, in financially wealthy communities, monitoring by volunteer wardens at nature reserves and by amateur naturalists.

The third most distinct group is *Collaborative monitoring with external data interpretation* (category C). Scientists designed these systems and analyze the data, but the local stakeholders collect the data, make decisions on the basis of the findings, and carry out the management interventions emanating from the monitoring system. These programs include, for example, many Indigenous guardian programs (also known as indigenous rangers or watchmen or -women; Reed et al. 2020a), BirdLife International's monitoring of important bird areas, and fisher and hunter records programs such as wildlife triangle monitoring (Cretois et al. 2020).

The least distinct monitoring systems belong to the categories *Externally driven monitoring with local data collectors* and *Scientist-executed monitoring* (categories D and E). In category D, local stakeholders are involved in data collection but other activities are carried out by professional scientists. Example programs within monitoring category D are volunteer monitoring of water or air quality, sound, rainfall, weather, vegetation, fungi, mammals, birds, amphibians, fish (for a review, see Kelly et al. 2020), invertebrates, bacteria, invasive species, and toxic algal blooms (Anderson et al. 2019) and fisher, angler, and hunter records programs, and data collection by paid local people.

In monitoring systems of category E, all aspects of the monitoring are undertaken by professional scientists, with no involvement of local stakeholders. The five categories are not sharply defined, and hybrid models exist (the white leaves in figure 2b).

The routes to feelings of ownership of a natural resource management process (figure 1d) all increase in importance across this spectrum of natural resource monitoring approaches. Early involvement in the design, especially when the monitoring systems address issues that are priorities for communities, lead to much stronger engagement of some segments of communities, including community leaders. We consider monitoring systems of the three most participatory categories (A, B, and C) to be locally based approaches to monitoring (for definitions of key terms, see the glossary in Eicken et al. 2021 [this issue]). Within these three categories, the power balance between local and external interests shift between the B and C categories—that is, whether data interpretation is undertaken by locals or by external stakeholders (Fry 2014).

The continuum of monitoring approaches mirrors the devolution of management responsibility in different approaches to natural resource management (Danielsen et al. 2009). The most local monitoring approach (category A) is typically part of customary systems of conservation management, whereas the least locally based of the monitoring approaches (category E and partly category D) parallel those conservation approaches that do not involve local people and in which the majority of decisions are made by remote government agencies or nongovernmental organizations (NGOs). The monitoring approaches between these two extremes (categories B, C, and partly D) mirror community-based and collaborative resource management with different roles of community members and government agencies and NGOs in sharing of management costs and benefits.

How does this spectrum connect with the different models of citizen science? Citizen science projects can be characterized by project goals (e.g., Wiggins and Crowston 2011, Haklay 2012) or according to the involvement of the public in the collection, interpretation, or entire survey process (Bonney et al. 2009, peer-reviewed version in Shirk et al. 2012). The monitoring systems of category D are mainly contributory, whereas those of categories B and C are mainly collaborative or cocreated.

The spectrum of monitoring approaches has been useful to the development of the practice of monitoring. It has nuanced the discussions of the benefits and disadvantages of locally based natural resource monitoring and that executed by professional researchers. One approach is not more optimal than the other; the chosen monitoring approach should depend on the context of the monitoring and the expected outcome. With this understanding of what locally based monitoring is, we will now explore the kinds of data it can generate and what the data can be used for.

## What kinds of data can locally based monitoring generate?

Locally based monitoring can collect data on the status and trends of the Earth's natural resources, ecosystem services, and species. These data are important because there is a serious risk that because of limited data currently available, especially in remote areas (Metcalf et al. 2018), natural resource management decisions are poorly targeted at addressing the most critical actions.

### Comparing community-collected data

We have been primarily interested in the use of monitoring data for decision-making. As such it has been important to understand if local approaches to natural resource monitoring can provide high-quality data (Parry and Peres 2015, Beirne et al. 2019). If monitoring by local communities is inaccurate or biased, locally based monitoring may not be reliable for assessing natural resource trends, and management interventions may be directed inappropriately (McKelvey et al. 2008, Game et al. 2018). We were able to examine this issue by completing five separate tests, using broadly accepted data collection techniques by scientists as the yardstick (discussed in box 1). These tests showed that community members and scientists produce closely similar results across a range of monitoring methods and types of natural resources monitored.

Box 1. **Toward robust comparative data quality studies.**

Addressing whether local people are able to produce high-quality data for decision-making is in some senses uninteresting (Wheeler et al. 2020). If local people are trained, the data collection is not technically challenging, or they are collecting information on elements of the natural world that they know well, there should be no *a priori* reason why they should not produce data sets as good as the data produced by scientists and field assistants. However, given the skepticism in some scientific communities about whether this is true, robust studies to address this question will probably continue to be important (Temple et al. 2020).

In our experience, five topics in comparative data quality studies merit special attention but are often overlooked.

First, many studies implicitly assume the superiority of the scientist-collected data set (Specht and Lewandowski 2018). This may be understandable, because the scientist-collected data sets often represent the broadly accepted techniques. However, scientist-executed methods also have weaknesses. For example in Australia, it has been shown that when Indigenous community members and scientists surveyed a population of lizards, they observed different segments of the lizard population (Ward-Fear et al. 2019). The community members detected lizards that were shyer and more difficult to see than were those detected by the scientists, suggesting that one of the groups’ sampling was unrepresentative. Comparative studies should question the quality of both groups’ data sets, and any assumptions about superiority of one or the other data set should be made explicit.

Second, without a baseline of truth against which to compare scientific and locally produced data sets, comparisons often merely become tests of the similarity of the approaches, rather than of the objective truth of either.

Third, comparative data quality studies often are undertaken without proper consideration of coverage and scale. For example, published studies that have reported contradictions between community members and scientists had mismatches between the temporal and spatial scales, timing, or geographical area, and the mismatches are likely to have influenced the comparisons (Danielsen et al. 2014a).

Fourth, the classification of the people involved is rarely carefully documented. A scientist may cover a relatively untrained or inexperienced person (e.g., a young PhD student) as well as longstanding expert in the biodiversity of the region. The same is true for community members.

Finally, there is a learning curve for community members as well as for scientists (Fox et al. 2017). Therefore, comparative studies undertaken in parallel with the establishment of a monitoring program may not provide meaningful outcomes.

**Figure 3.**
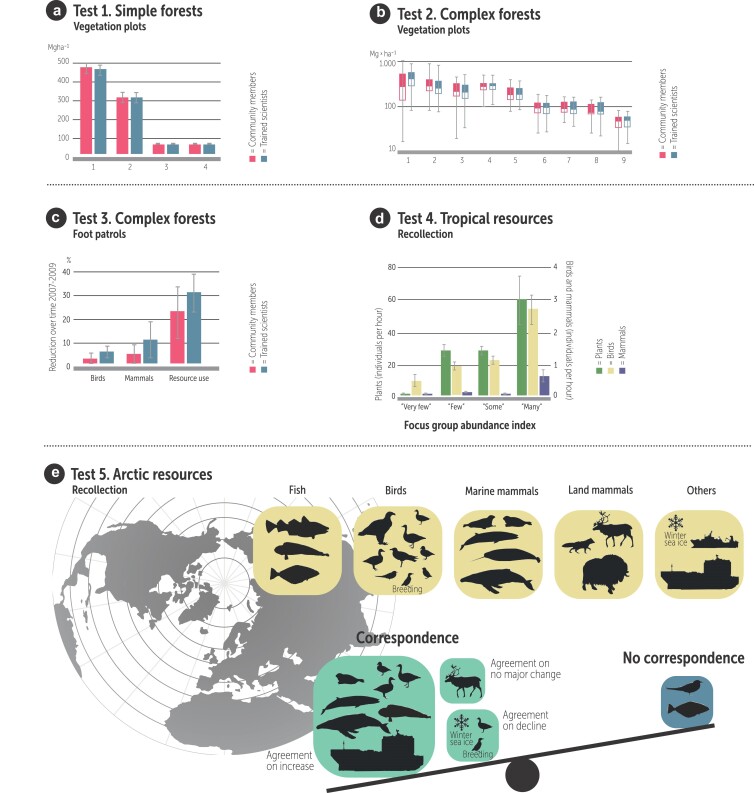
Examples of the data that locally based monitoring can generate. The results of five separate tests of the quality of data from community members using vegetation plots in simple and complex forests (a), (b), foot patrols in forests (c), and recollection through focus group discussions of tropical (d) and Arctic resources (e). (a) Test 1 shows measurements of woody biomass by community members and scientists over a range of forests in India (sites 1–3) and Tanzania (site 4). (b) Test 2 shows measurements of woody biomass by community members and scientists over a range of forests in Indonesia (site 1), China (sites 2–3), Laos (sites 4–5), and Vietnam (sites 6–9; n = 289 permanent plots; log10 scale indicating the smallest observation, lower quartile, median, upper quartile, and largest observation). (c) Test 3 shows the reduction percentage of 68 forest resources and forest uses recorded by community members and scientists over 2.5 years in Madagascar, Nicaragua, the Philippines, and Tanzania (n = 300 pairs of observations; each paired observation represents a time series of parallel records of sequential quarterly registrations of one resource or resource-use event at one site by community members and scientists). (d) Test 4 shows the relationship between community member focus group statements of abundance of plants, birds, and mammals and the average abundance indices (the number of individuals observed per hour) of the same resources obtained by community members’ and scientists’ independent transect walks between 2007 and 2009 at nine study sites in Bosawás Biosphere Reserve, Nicaragua. (e) Test 5 shows the attributes for which community members in Disko Bay, Greenland provided abundance information (top) and the correspondence with scientists’ assessments (bottom). Community members provided information on the abundance of minke whale (Balaenoptera acutorostrata) in two areas, but in the upper panel we show the species only once. From Danielsen and colleagues (2011, 2013, 2014a, 2014b, 2014d), including the online data sets for panels (c)–(e).

Our first two tests assessed how well community members could assess forest aboveground biomass (carbon stocks) using vegetation plots in simple (figure 3a) and complex forests (figure 3b). The amount of carbon stored in forests is important because carbon dioxide is the primary greenhouse gas emitted by human activities, and changes in forest carbon can help to mitigate climate change, or they can exacerbate the problem. In figure 3a and 3b, measurements by community members are shown in red and those by scientists are shown in blue. In both simple and complex forests, local communities collected information on forest biomass of comparable quality to scientists (Danielsen et al. 2011, 2013).

Our third test examined how well community members could assess natural resources (birds and mammals) and resource use with foot patrols. Forests resources are important because about 240 million people live in tropical forests (Peskett et al. 2008), including some of the world's poorest and most marginalized communities, who use the forest as their resource base (Funder 2009). Figure 3c shows the trend over time (reduction percentage) of 68 forest resources and forest uses (snares, fires, etc.) recorded by community members and scientists. The community members used regular foot patrols and the scientists surveyed along fixed routes within the same forest study sites using a variable distance line transect method (Fragoso et al. 2016). Despite considerable differences between countries, cultures, and types of natural resource monitored, community members and scientists produced closely similar quantitative results on status of and trends in the abundance of species and natural resources (Danielsen et al. 2014a).

In our fourth and fifth tests, we explored how well community members could assess natural resources using recollection. These two tests were undertaken in environments that are relatively inaccessible to scientists, in the tropics (figure 3d) and the Arctic (figure 3e). In the fourth test, one set of data came from community members’ natural resource abundance data collected through recollection in community focus groups on the basis of conclusions drawn from general use of natural resources. The other data set came from other community members’ and scientists’ line transects. Figure 3d shows the relationship between community member focus groups’ statements of abundance of plants, birds, and mammals and the average abundance indices (the number of individuals observed per hour) of the same resources obtained by community members’ and scientists’ transect walks. The community members’ recollection of the abundance of forest resources compared well with the line transect results.

In the fifth test, we compared community members’ recollection with reports in the scientific literature of trends in the abundance of 24 natural resources in the Arctic. Arctic living resources are of great importance to the livelihood, culture, and subsistence economies of Indigenous and local communities (Nuttall 2018). Figure 3e shows the attributes that community members provided abundance information about (top) and the correspondence with scientists’ assessments (bottom). The community members and the scientists produced corresponding results for 12 attributes. Only for two populations, nearshore Greenland halibut *Reinhardtius hippoglossoides* and breeding Arctic tern *Sterna paradisaea*, did community members’ and scientists’ reports disagree. For ten attributes, we were unable to locate any scientist-produced data in the published literature to allow for a comparison with the community members’ findings.

In all five tests, the community members used scientific methods for data collection; they did not develop their own techniques or employ autonomous indicators (Sheil et al. 2015). Moreover, in two of the five tests, the local monitoring programs were established experimentally for the purpose of the tests and they were not running independently, outside of the research context, at the time of the test. Despite these weaknesses, our five tests across a range of ecosystems and sociopolitical settings suggest that locally based approaches are capable of providing accurate and precise information independent of external experts. Our findings concur with previous studies in the same habitats when there were no differences in scale, place, and time of the survey effort by community members and scientists (box 1). Approaches for optimizing sampling accuracy are discussed in box 2.

Box 2. **Optimizing accuracy of sampling protocols.**

**Figure 4.**
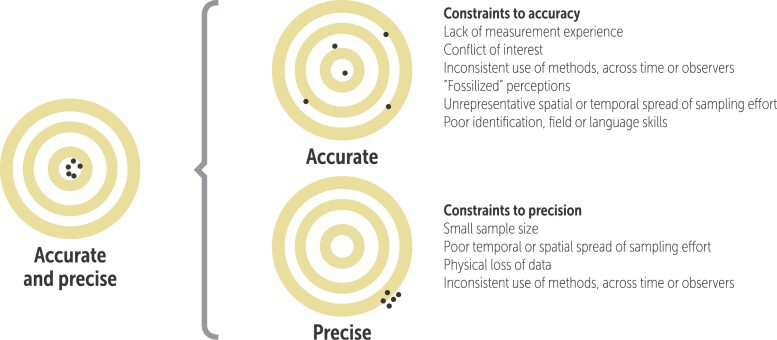
Key potential constraints to the accuracy and precision of natural resource monitoring programs. The constraints apply to all monitoring programs, whether they are undertaken by scientists or citizens (modified from Danielsen et al. 2005a).

Considerations of accuracy, precision, and overall utility of locally based monitoring programs are all best addressed by careful planning (Danielsen et al. 2005a, Sherbinin et al. 2021). Several potential constraints to the accuracy and precision apply to all natural resource monitoring programs, whether they are undertaken by scientists and community members (figure 4), and paying attention to these constraints may be helpful for studies to address and mitigate biases.

In situations in which funding is available, and the apparent abundance of natural resources allows regulated harvesting or financial payments to communities, local communities may have an incentive to report false positive trends in those natural resources so that they can continue to harvest the resources or to be paid, even though the natural resources actually may be declining (Nielsen and Lund 2012, Lund 2014). Periodic triangulation of the monitoring results will therefore be required (Danielsen et al. 2011, Flick 2018). Triangulation can also overcome the bias that results from single method, single observer, and single data source studies by using multiple observers, methods, and data sources (Denzin 1978). Often the organizers of monitoring programs can undertake triangulation across communities, community members, and methods. Triangulation can, for instance, be based on random spot checks in which a subset of the area is resampled using other monitors or other field methods (e.g., remote sensing of forest cover). It can also be combined either with allocating individuals with rank based on their knowledge, allowing data to be disaggregated according to this ranking, or with a statistical analysis of the community-based data to search for anomalies or trends that are beyond the normal or expected range.

Among the methods used in locally based monitoring programs, recollection through focus group discussion is one of the most cost effective in generating natural resource management interventions (Danielsen et al. 2007). Perception-based information is, however, memorized and therefore sometimes lost (Thurstan et al. 2016); although in communities mainly using verbal history, collective memory is often well remembered. Key measures that can be taken in focus group discussions to reduce biases beyond triangulation include increasing the number of primary data providers, using unequivocal categories of resource abundance, and ensuring that the moderator has skills and experience in facilitating dialogue. Such measures are easy to undertake even in modestly funded monitoring initiatives. New approaches to the systematic collection of views and judgments in decision-making, such as the Delphi and the nominal group technique method, may also be valuable (Hugé and Mukherjee 2018).

## What can data from locally based monitoring be used for?

We turn now to what locally based monitoring data can be used for in terms of decision-making and management (Villaseñor et al. 2016, Newman et al. 2017, Chase and Levine 2018, Fulton et al. 2018). The efficiency of decision-making is difficult to measure by a standard scale across different habitats, and the true impact may be discernible only in the long term. We therefore used natural resource management interventions as a proxy for decision-making.

We defined a natural resource management intervention as a purposeful action by a managing body to change the use of natural resources. Examples include the development of bylaws at village and municipal level, local time and area closures for natural resource extraction, and restrictions on specific resource extraction methods and gear. We were able to study natural resource management interventions in locally based monitoring systems established, and running outside of a research context, in Greenland, Tanzania, and the Philippines, all in the monitoring category *Collaborative monitoring with local data interpretation* (category B, figure 2). Within the first 3 years of operation, the locally based monitoring system in Greenland led to 14 distinct recommendations for 12 natural resources in the four communities that were involved (Danielsen et al. 2014d). These proposals related, for instance, to conservation of marine habitat, influencing marine harvest techniques, and influencing goose harvest pressure, and they would require action by the community members themselves, the local municipal authority (e.g., municipal bylaws), the central government (e.g., the setting of quotas), or by other institutions.

Likewise, in a locally based natural resource monitoring system in Tanzania, 181 natural resource management interventions were proposed by the community members as a result of the monitoring after just 10 months of operation in 23 communities (Topp-Jørgensen et al. 2005, Danielsen et al. 2010b, Nyamoga and Ngaga 2016). A total of 21 of the 23 communities had suggested management interventions that targeted what scientists had independently identified as the most serious threats to their respective forests. These threats were wood extraction in lowland miombo woodlands and hunting and fire in montane evergreen forests. Of the interventions targeting the most serious threats, 75% had been implemented by the main organizing bodies for forest management in the communities, the Village Natural Resource Committees, at the time of the assessment, whereas most of the others were awaiting approval by the district authorities.

**Figure 5.**
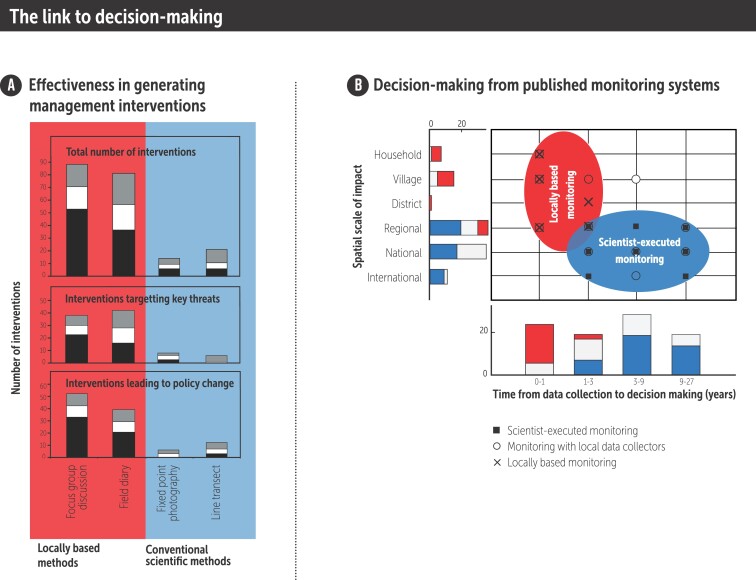
The link from monitoring to decision-making. Effectiveness of locally based and scientist-executed monitoring methods in generating natural resource management interventions in Philippine protected areas (a) and the decision-making from monitoring based on data from published monitoring systems (b). Left side (a) shows the effectiveness of locally based and conventional scientific monitoring methods in generating natural resource management interventions intended to improve the way that local people (black), outsiders (white), and both (grey) manage Philippine protected-area resources (n = 156 natural resource management interventions). The upper panel shows the total number of interventions generated by each method. The central panel shows the number of interventions that targeted the three most serious threats to the biodiversity of each site, and the lower panel shows the number of interventions that led to policy change within government or community institutions. Right side (b) shows decision-making from monitoring based on data from published monitoring systems (n = 104; n = 45 for the scientist-executed monitoring systems, n = 37 for the monitoring systems with local data collectors, n = 22 for the locally based monitoring systems). The circles include all the scientist-executed (blue) and all the locally based monitoring (red) systems. The bar chart indicates the number of scientist-executed monitoring systems (blue bars), monitoring systems with local data collectors (white bars) and locally based monitoring systems (red bars) at each level of spatial scale and implementation time. From Danielsen and colleagues (2005b, 2007, 2010a), including the online data sets.

Although these examples strongly suggest that locally based approaches to natural resource monitoring can be effective in generating natural resource management interventions, they do not show how these systems would compare with conventional monitoring approaches without community involvement. We were however able to compare the return on investment in terms of natural resource management interventions of locally based and conventional biodiversity monitoring methods in eight Philippine protected areas (Danielsen et al. 2007). The two sets of monitoring approaches were compared across 1.1 million hectares of protected areas. One set included two methods that included 350 local people in data collection and discussion of data. These methods were the focus group discussion method and the field diary method. The other set of methods was composed of standardized techniques that follow normal scientific requirements for objectivity and repeatability. These methods were fixed-point photography and the line transect method. The methods were carried out by protected-area staff without the involvement of local communities. Both sets of monitoring approaches received orientation and financial support from the same externally funded, government-led project. Natural resource management interventions undertaken over a 2 year and 7 month period were included. Before the two sets of monitoring approaches were established, the environmental monitoring activity of the protected area staff was restricted to assessment of extracted timber. Very few, if any, management interventions emanated from this. Figure 5a shows the effectiveness of locally based and conventional scientific monitoring methods in generating natural resource management interventions intended to improve the way local people (black), outsiders (white), and both (grey) manage Philippine protected-area resources. The upper panel shows the total number of interventions generated by each method. The central panel shows the number of interventions that targeted the three most serious threats to the biodiversity of each site, and the lower panel shows the number of interventions that led to policy change within government or community institutions. We found that, for approximately the same recurrent government investment, far more interventions result from locally based biodiversity monitoring methods than conventional scientific ones (figure 5a, upper panel; *n* = 156 natural resource management interventions). This pattern also holds if we restrict the analysis to those interventions that target only the three most serious threats to species populations and habitats at each site (figure 5a, central panel). Moreover, if we look at interventions that led to policy change with a potential long-term impact on sustainable development (i.e., new resolutions or bylaws), the same pattern emerges (figure 5a, lower panel). This general difference held true whether we considered resources used by local people or by outsiders (figure 5a). Our data suggest that locally based monitoring cost-effectively generates natural resource management interventions not only to address resource use by locals but also by people from other places.

These results may, however, be context dependent and not applicable in other countries. To examine the generalizability of our findings, we undertook a meta-analysis of published natural resource monitoring systems to assess whether participation in data collection and analysis in monitoring systems influenced the speed and scale of decision-making and action. We reviewed approximately 3400 papers presenting published monitoring results. We found that the degree of involvement by local stakeholders in natural resource monitoring profoundly influences the spatial scale and speed of decision-making based on the monitoring data (figure 5b). The greater the involvement of local people in monitoring activities, the shorter the time it takes from data collection to decision-making following monitoring. Two types of participatory monitoring are recognized: one in which local people collect data but the interpretation is done by someone else (categories C and D in the spectrum of natural resource monitoring systems in figure 2) and another in which local people collect and interpret the data themselves (categories A and B monitoring systems). The most local and participatory of these two options leads to management decisions, which are typically taken at least three to nine times faster than scientist-executed monitoring, although they operate at much smaller spatial scales (figure 5b). Scientist-executed monitoring informs decisions in regions (44%), nations (38%), and international conventions (18%; *n* = 45 scientist-executed systems).

Our findings from the meta-analysis suggest that locally based monitoring and professional monitoring lead to substantially different kinds of decisions (Danielsen et al. 2005a). Decisions from locally based monitoring are often taken promptly and at the local level, by local government agencies and community leaders, in response to immediate threats to the environment. These decisions often result in actions based on community rules and enforcement, such as local bylaws governing resource use (Wilson et al. 2018). Such actions are aimed both at protecting habitats or species and at ensuring a continued supply of benefits for the local communities (Danielsen et al. 2014c). The decisions are often respected by the locals and the associated actions are relatively sustainable, both financially and organizationally (Danielsen et al. 2005b), probably because they are nested within existing, often local institutions. For instance, a community monitoring group reported a decline in the abundance of marine fish in a bay in the Philippines. In response, the municipality issued an ordinance allowing fishing with hook and line but banning the use of nets and compressors in the bay. The ordinance was widely respected by the locals, and after only 7 months the abundance of fish in the bay reportedly increased.

This kind of monitoring generally provides fast and meaningful feedback to inform adaptive management (Allen and Garmestani 2015, Schemmel et al. 2016). In comparison, monitoring by scientists may be slow in leading to decisions but the scale of decision may be very different (Danielsen et al. 2005a). Professional monitoring has the potential to influence national and global policies and funding flows. Scientists often have better access to high-level decision-makers than local communities. For instance, results from locally based monitoring are unlikely to persuade the US government to ratify the Kyoto Protocol, whereas findings from professional monitoring potentially could. Locally based monitoring is generally simple; it rarely provides the body of evidence (controlling for all kinds of confounding factors) that will impress those who develop national and international regulations. If a decision for legal or jurisdictional reasons must be made at national level, then rapid decision-making by local peoples may not always be helpful. Decisions from locally based monitoring can however have impacts beyond the local scale when the locally based monitoring is embedded within or linked to a national or international scheme that feeds the data up to the levels at which governments, international agencies, and multinational corporations operate (Danielsen et al. 2014c, 2014d, Pocock et al. 2018, Eicken et al. 2021 [in this issue]). Whereas monitoring of macro scale (global) environmental changes is usually undertaken by scientists, community members often monitor how large-scale environmental changes play out at the local level (for instance changes in the abundance and composition of sea ice in the Arctic; Eicken et al. 2021 [this issue]). Community members also can attend international policymaking processes together with scientists and provide their perspectives on the environment and the actions that need to be taken.

We suggest three reasons why locally based monitoring leads to decision-making (Danielsen et al. 2005a). First, unlike monitoring by scientists, locally based monitoring can encourage decision-making by providing an institution (e.g., village discussion groups composed of particularly knowledgeable villagers) or a forum (e.g., meetings between rangers and local residents) for regular discussion of local natural resource management. Monitoring provides a reason to discuss better ways of managing resources. Second, locally based monitoring can provide local residents with otherwise rare opportunities for collaboration with government staff and for representation in local decision-making on natural resources. Understanding the local ways of thinking, and of making decisions, is therefore important for the success of local systems. Third, decision-making based on local monitoring may not be swamped by government bureaucracy, because many of the decisions are taken promptly by the same people or institutions that collect the data (Danielsen et al. 2005b).

Our findings suggest that promptness of decisions emanating from monitoring is a characteristic of locally based approaches to natural resource monitoring. The general pattern we found in the Philippines also held true when we looked at published monitoring systems from across the world. Our literature analysis suggested that decisions from locally based approaches tend to be taken at the local, operational levels of resource management, where they involve the people who face the daily consequences of environmental changes.

## Can locally based monitoring empower people in natural resource management?

Following our exploration of the link from local monitoring to decision-making, we investigated the potential to empower local people in natural resource management. We know of only three studies of the local perceptions of monitoring and the factors promoting local uptake of monitoring (Turreira-García et al. 2018). The first one, in Tanzania, provides preliminary evidence of political, social, and economic empowerment. The other two, in the Philippines and in Greenland, add to this evidence, but much more research is needed.

**Figure 6.**
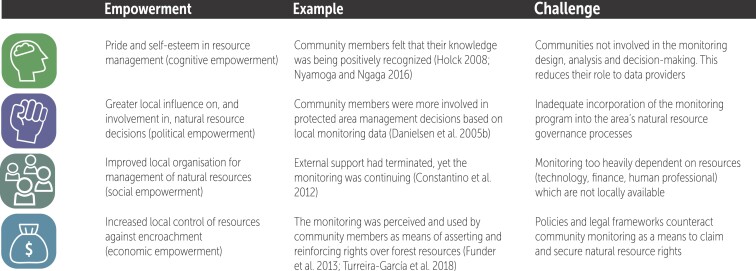
Overview of cognitive, political, social and economic empowerment of community members in natural resource management from locally-based monitoring, including examples as well as potential challenges involved.

We examined three category B monitoring systems (cf. figure 2) that all were established outside of a research context. We defined empowerment as a participatory, developmental process through which individuals and groups gain greater influence over their lives and acquire improved control over valued natural resources (modified from Maton 2008). Empowerment can occur in different dimensions, visualized with icons in figure 6. These include cognitive (e.g., development of pride and self-esteem in natural resource management), political, social, and economic (Speer and Hughey 1995, Maton 2008, Constantino et al. 2012).

In Tanzania, we led a study of community member responses to a natural resource monitoring system established in 23 villages (Funder et al. 2013). This monitoring system aimed at providing information on the status and trends of forest use and forest resources, for use by communities in daily forest management in participatory forest management areas (Topp-Jørgensen et al. 2005). Shortly after its establishment, the monitoring system was left for villagers to continue (Nyamoga and Ngaga 2016). We returned to the area 4 years later and found that the monitoring system was continuing to operate in most of the villages. Moreover, in four villages covered by a household survey, 86% of the respondents said they felt the monitoring system benefitted their household (*n* = 160 households).

This broad ownership to and local support for the monitoring system and its continuation (entirely without external assistance) prompted search for an explanation. When asked directly to name the benefits of the monitoring system, all of the 138 respondents that were favorable to the system mentioned protection against encroachment on forest resources as the main benefit.

For example, a respondent from one village stated “It shows them (i.e., people from other communities) that the forest belongs to us.” In that village the monitors had used the information collected through monitoring as an argument in their negotiations with the neighboring villages, by claiming that they are the ones who know the forest and its conditions best, and that they therefore are its best custodians. It was therefore not only the physical presence of the monitors in the forest but also the data and the act of collection that mattered. Community members placed great emphasis on how the monitoring system supported individual and collective access to and control over resources, thereby ensuring long-term access to natural ecosystem products and services. The monitoring system was therefore perceived and used by community members as a means of asserting and reinforcing rights over forest resources.

Our findings suggest that the monitoring system has provided an organizational and political arena for community empowerment. The scientific basis for the monitoring was an important element in the community empowerment, by producing empirical data that government found hard to ignore (Funder et al. 2013). The opportunities that the monitoring system provided for community members did not, however, always play out equitably within communities. The monitors were generally better-educated crop-producing farmers from the better-off segments of the villages. Some of them viewed the hunting by minority groups of pastoralists as primitive and destructive, and they opposed the pastoralists’ interest in forest management referring to information obtained through the monitoring system on the community's forest use. Further work is needed to explore how elite capture of the monitoring process can be avoided (Lund et al. 2018).

Our findings provide preliminary evidence that when locally based monitoring is an integrated part of the natural resource governance system, the monitoring can be a very important mechanism for empowerment of the community members (Funder et al. 2013, see also Quintana et al. 2020). In particular, in terms of *political empowerment*, the application and generation of local knowledge in the monitoring system led to greater local influence on and involvement in natural resource management decisions (figure 6, left column). In terms o*f social empowerment,* the monitoring led to improved local organizations for management of resources. In terms of *economic empowerment*, the monitoring led to increased local control of valuable subsistence forest resources vis-à-vis the state and other communities. Locally based monitoring can therefore contribute to create and reinforce local and ethnic identity (Reed et al. 2020b).

In the two other studies in which we assessed the local perceptions of locally based monitoring, we found further evidence of increased local empowerment. In the Philippines, Indigenous zoning and resource-use regulation systems such as protection of sacred streams were reestablished with government recognition as a result of locally based monitoring. Moreover, the Indigenous community members were increasingly being recognized by the local government staff as resource comanagers (Danielsen et al. 2005b). We likewise observed in Greenland that a primary reason for the interest in locally based natural resource monitoring among community members stems from the fact that enrolling in the monitoring provides an opportunity for the participants’ insights and knowledge on natural resources to inform government decisions, thereby enabling their voices to be heard (Danielsen et al. 2014d). In figure 6, we provide further examples and we summarize the key potential challenges.

The process aspect of locally based natural resource monitoring systems is very important to the community members’ sense of empowerment (Danielsen et al. 2014b). The most profound empowerment is likely to be achieved when community members themselves are the gate keepers, detecting and deciding which data are complete and which are false, or which are out of context and therefore need to be discarded. Community members’ ownership of the data and information and their control over the knowledge, the validation process, and the application of the knowledge are critical (Huntington 2011).

## Where is locally based monitoring suitable?

Locally based natural resource monitoring has been demonstrated as being suitable for monitoring organisms or phenomena that are meaningful to community members—for example, as a source of food or income or because they are of sociocultural value. Locally based monitoring can help obtain a more complete knowledge of the changes and status of natural resources, particularly where scientist monitoring is sparse or too expensive to sustain and for ecosystem attributes where remote sensing cannot provide credible data (Hollings et al. 2018, Beirne et al. 2019). However, if the aim is to monitor attributes that are not relevant from a local perspective, locally based natural resource monitoring may not be suitable (de Mattos Vieira et al. 2015). In some contexts it also may require a strong effort to obtain trust and understanding about how monitoring can be used positively to ensure a better future. In many areas, the alternative to local systems would be no monitoring at all.

**Figure 7.**
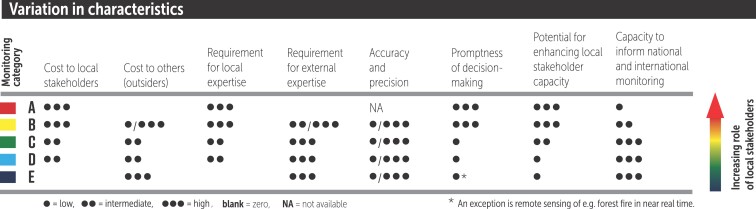
The variation in eight key characteristics across the spectrum of five categories of monitoring systems (Danielsen et al. 2009). Note that exceptions are common. Monitoring categories are defined in figure 2.

**Figure 8.**
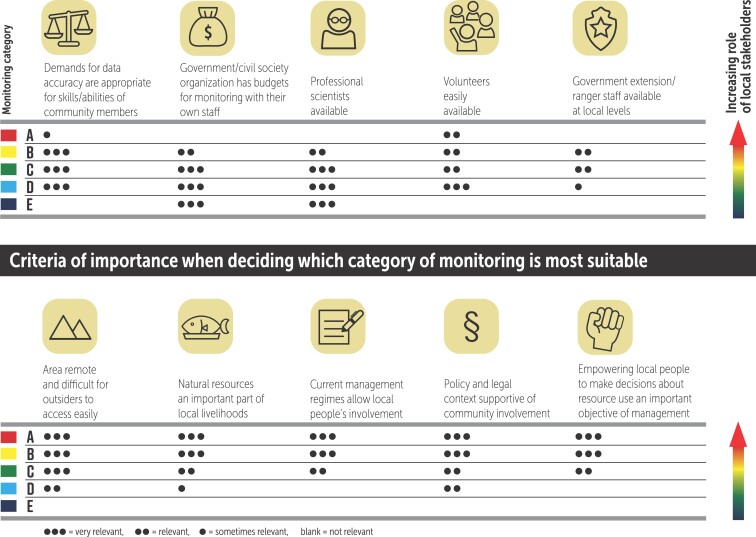
Criteria of importance when making decisions on which category of natural resource monitoring system is most suitable for a given circumstance. The relative role of local stakeholders in the monitoring systems increases from bottom to top between the five categories of monitoring systems (Danielsen et al. 2009). Monitoring categories are defined in figure 2.

When choosing a category for a monitoring system, the context and aims of the initiative will therefore define which systems are most appropriate. To help choose a suitable category, we have prepared tables showing the variation in key characteristics across the categories of monitoring systems (figure 7) and the criteria of importance when deciding which category of monitoring is suitable (figure 8). The tables present general guidance based on the expertise of the authors, and individual monitoring programs can vary substantially from the pattern described.

*Scientist-executed monitoring* (category E) systems can be executed almost anywhere because they are largely externally driven (figure 8). They are most suitable where highly technical monitoring across large scales demands high levels of professional expertise and where there is a guaranteed source of funding that will permit this form of technical monitoring to be sustained over time.

*Externally driven monitoring with local data collectors* (category D) can be used where there are skilled volunteers or funds to pay staff for the collection of field data (figure 8). This type of system is ideal when large numbers of people are required to collect data across wide geographical areas and on a regular basis. This capitalizes on the strength of gathering the most data possible, even if the accuracy or precision of each individual data point may be less than that obtained by highly trained professionals.

*Collaborative monitoring with local* (category B) and *external* (category C) *data interpretation* depends on local people making a significant investment in monitoring. These systems are therefore most appropriate where local people have a significant interest in natural resource use and ecosystem services, when the information generated can have an impact on how the resources can be managed and the monitoring integrated within the existing management regimes, and when there are policies or strategies in place that enable decision-making at the local level (figure 8). In these systems, local ownership, empowerment, and a link to management decisions can be even more important than data quality (figure 7), although management and empowerment benefits should not be used as an excuse for poor design (Danielsen et al. 2009). A balance is therefore needed between monitoring goals and the broader goals of seeing the results used and decisions taken about natural resource management. *Autonomous local monitoring* (category A) systems, by definition, cannot be instigated from outside.

## How can locally based monitoring evolve into the future?

Our review shows that natural resource monitoring anchored in and conducted by communities can deliver credible data, inform decision-making, and empower communities for resource governance. The concept of natural resource monitoring departs from a *command-and-control* culture of managing natural resources (Holling and Meffe 1996). Instead, the concept of locally based monitoring comes from a democratic*, community-driven* culture, where the emphasis is on inclusive approaches, comanagement and community-driven conservation.

Locally based approaches provide strong incentives for resource users to engage in monitoring as a tool for long-term management (see figure 1c, 1d). The extent to which local monitoring leads to sustainable resource use however probably also depends on broader aspects of how resources are governed. Most importantly, this includes inclusion of community members in decision-making over the actual management of the resources, ensuring that communities feel secure in their rights to benefit from resources (in a sustainable way), and making sure that communities’ own organization around monitoring and resource use is representative and transparent (Pagdee et al. 2006, Porter-Bolland et al. 2012).

Communities that use the environment on a daily basis represent a vast but largely untapped source of knowledge on the world's environment. If we are to unlock the full potential of people-based environmental observations to yield new knowledge about life on Earth, as well as to guide evidence-based decisions, we need further understanding of how to obtain and use data from different people (with varying beliefs, epistemologies, rationalities, and cosmologies) and different knowledge systems. For example, we need to find out when (at which step in the monitoring process), how (in which way), and by whom (the organizers of the locally based monitoring programs, the community members, or the scientists) the findings and observations from locally based monitoring can be robustly connected with scientist-executed monitoring to inform decisions on resource management.

### Providing a path for knowledge to travel both upward and downward

An important future development in locally based monitoring will be to find ways to connect monitoring across scales (Luzar et al. 2011, Evans et al. 2018). International organizations managing global data sets on the environment (forest cover, protected areas, threatened species, key biodiversity areas; Eken et al. 2004) strive but generally fail to make their data sets of a temporal and spatial resolution suitable for local decision-making. These data sets are typically updated annually or monthly, and they rarely have an effective grain size appropriate for landowner decisions at the level of a single field or crop. Likewise, observations by community members rarely form part of global data sets. Locally based monitoring might, however, provide a path for local knowledge on the environment (including Indigenous knowledge, Tengö et al. 2021 [this issue], and Local Ecological Knowledge) to travel upward to higher levels and, at the same time, enable the global data sets to obtain a resolution useful for guiding action by local stakeholders.

Valuable lessons could be learned from the eBird, iNaturalist, and Open Data Kit programs (Brunette et al. 2017). In the latter, environmental data entered on smartphones are sent to a server, which presents graphs in the office or on a website in real time. Provided that such data are also discussed, analyzed, and ideally co-owned with communities, they could potentially be useful both locally and at national and international levels. All three programs do, however, rely on smartphones (Andrachuk et al. 2019). Many community members do not use phones, particularly in the often remote, low-technology, and difficult environments in which frontline natural resource management actions unfold. It is crucial to identify robust ways for local knowledge to travel upward in these contexts.

### Connecting with technology-reliant monitoring

Over the past decades, the practice of environmental monitoring has moved toward an increasing dependence on large technology companies, specialized expertise, and vast processing power for collecting, analyzing, and using data. Fast-growing technologies within environmental monitoring include e-DNA, automated voice and image recognition, drones and remotely operated underwater vehicles, tracking of individuals, remote sensing, artificial intelligence (e.g., the Global Fishing Watch, www.globalfishingwatch.org), web scraping from social media (Toivonen et al. 2019), and advanced statistics (Pimm et al. 2015). This includes many approaches where there is no role for community members or where their role is limited to data registration. This can reduce communities and, more broadly, citizens to the role of spectators, thereby preventing some of the key benefits of citizen participation and locally based monitoring from being realized. Community members may in a few special cases contract scientists to monitor for them, but their own ability to lead and undertake technology-reliant monitoring may be limited.

It is important to create a strong link between local approaches and the evolving technological advances in monitoring so that these advances benefit community members and promote sound resource management (Johnson et al. 2021 [this issue]). Fortunately, there are many opportunities for doing so. Remote sensing imagery, using locally based data for ground-truthing, can enable community-based organizations to map forests in real time, thereby adding value to the locally based monitoring of their territories (Chapin et al. 2005, Schepaschenko et al. 2015, https://explorer.naturemap.earth/about). Even demographic models, usually the domain of ecological modelers, can be transformed into easily accessible tools, populated with data from locally based monitoring and used by nonprofessionals. A harvest calculator can, for example, enable community members—independently from scientists—to undertake multiannual harvest planning of muskoxen (*Ovibos moschatus*) stocks, ensuring both a continued supply of meat for subsistence and of old bulls for guided trophy hunting (Cuyler et al. 2020). The calculator models future population levels of each herd, including uncertainty, on the basis of abundance data and assumptions regarding future harvest levels and demographic rates. If technology-based monitoring approaches are to make a real difference, community members must be engaged. At the same time, the more their data are used, the more the value of locally based monitoring will increase for the community members.

### Documenting sustainable production

Locally based monitoring could be developed further in three other areas. The first is for documenting environmentally and socially sustainable production as part of certification processes and eco labeling. Rural communities engaged in locally based monitoring could sell their products, such as meat, fur, agricultural commodities, handicrafts, and tourism, at higher prices if the products were labeled such that consumers know that purchasing the product is contributing to sustainable development and securing the rights of marginalized producers. Today, global data sets are used (https://bettercotton.org; Koval and Cervenicky 2020) but locally based monitoring could be an alternative low-cost and transparent approach to document this.

### Third-party monitoring in areas with travel restrictions

Second, locally based monitoring could be developed further as a tool for third-party monitoring (Niu et al. 2020). An increasing number of countries have long-standing travel restrictions (the Sahel region, Yemen, Afghanistan), including many areas that are hotspots for biological diversity. Organizations providing support for the environment and development need to know whether the initiatives funded are making progress. Locally based monitoring could be used for monitoring project delivery and impact, for collecting contextual information, and for learning what does or does not work in programs and for sharing this with partners.

### Early warning for zoonotic diseases

A third area in which locally based monitoring could be developed further is for monitoring wildlife health, preventing epidemics emanating from wildlife disease reservoirs (Halliday et al. 2012). Locally based monitoring could form an important element of early warning systems for zoonotic diseases where prompt detection can prevent outbreaks. Zoonotic viruses infect people directly, often through handling of live primates, bats, and other wildlife or their meat (Dobson et al. 2020, Gibb et al. 2020). Wildlife epidemics may, in some cases, be associated with marked changes in animal behavior (rabies, chronic wasting disease) and mortality (avian influenza, plague *Yersinia pestis*). Such changes could work as proxies for wildlife health. For instance, in 2001–2003, hunter-based monitoring showed that wild animal ebola outbreaks began before each of five human ebola outbreaks in Central Africa (Rouquet et al. 2005). Twice the health authorities were alerted to an imminent risk for human outbreaks, weeks before they occurred. The world's wildlife disease reservoirs are concentrated in certain areas (UNEP-ILRI 2020). In these areas, locally based monitoring aimed at targeting outbreaks in their early stages could prevent the spread of disease and reduce human morbidity and mortality. Improved detection of local zoonotic foci could be used to restrict access and prevent disruption of focal transmission cycles. This could reduce the displacement of disease reservoirs by deforestation and their spread through wildlife trade from areas where zoonotic reservoirs pose a substantial danger to human health.

### Toward enhancing resource management

How can our findings be turned into meaningful action? Despite the fact that previous global conservation agendas, such as the United Nations Sustainable Development Goals (Fritz et al. 2019) and the Convention on Biological Diversity, have recognized the critical importance of involving Indigenous and local knowledge in sustainable development efforts (e.g., Aichi target 18), locally based natural resource monitoring remains largely absent from mainstream conservation practice. As the world prepares to consider new, post-2020 conservation targets (Visconti et al. 2019), we show that systematically involving local communities in monitoring the natural resources they depend on can provide the means to substantially improve outcomes. Crucially, our findings from two decades of tests, from the tropics to the poles, peer-reviewed, and published in leading international conservation science journals, suggest that substantial natural resource management gains ought to be attainable. Regrettably, the reward system for scientists is still skewed toward scientific publishing and implementing the scientific process rather than helping address real-world problems in a coproduction of knowledge setting (Tregoning 2018). Locally based monitoring practitioners report continued challenges in working with governments to operationalize or act on community observations in decision-making (Danielsen et al. 2020). Locally based monitoring is often ignored, to be undertaken and continued over time without supportive policies, funds, or organizational structures (PMMP 2015, Costa et al. 2018). Moreover, the indicators being developed for the new global conservation targets are not suitable for bringing in local data (www.cbd.int/sbstta/sbstta-24/post2020-monitoring-en.pdf). Overcoming these challenges will be difficult, but it is a small task compared with the enormous gains that can be made for the world's environment by leaving no one behind.

## Author Biographical

Finn Danielsen (fd@nordeco.dk), Martin Enghoff, and Michael K. Poulsen are affiliated with the Nordic Foundation for Development and Ecology, in Copenhagen, Denmark. Mikkel Funder is affiliated with the Danish Institute of International Studies, also in Copenhagen. Per M. Jensen is affiliated with the Faculty of Science at the University of Copenhagen. Neil D. Burgess is affiliated with the Centre for Macroecology, Evolution, and Climate at the University of Copenhagen and with United Nations Environment Programme's World Conservation Monitoring Centre, in Cambridge, United Kingdom.
